# Understanding the Impact of Omega-3 Rich Diet on the Gut Microbiota

**DOI:** 10.1155/2016/3089303

**Published:** 2016-03-14

**Authors:** Blanca S. Noriega, Marcos A. Sanchez-Gonzalez, Daria Salyakina, Jonathan Coffman

**Affiliations:** ^1^Division of Clinical & Translational Research, Larkin Community Hospital, South Miami, FL 33143, USA; ^2^Computational Biology and Bioinformatics, University of Miami, Coral Gables, FL, USA; ^3^Department of Infectious Disease and Response, Saba University School of Medicine, Saba, Dutch Caribbean, Netherlands

## Abstract

*Background*. Recently, the importance of the gut microbiota in the pathogenesis of several disorders has gained clinical interests. Among exogenous factors affecting gut microbiome, diet appears to have the largest effect. Fatty acids, especially omega-3 polyunsaturated, ameliorate a range of several diseases, including cardiometabolic and inflammatory and cancer. Fatty acids associated beneficial effects may be mediated, to an important extent, through changes in gut microbiota composition. We sought to understand the changes of the gut microbiota in response to an omega-3 rich diet.* Case Presentation.* This case study investigated changes of gut microbiota with an omega-3 rich diet. Fecal samples were collected from a 45-year-old male who consumed 600 mg of omega-3 daily for 14 days. After the intervention, species diversity was decreased, but several butyrate-producing bacteria increased. There was an important decrease in* Faecalibacterium prausnitzii* and* Akkermansia* spp. Gut microbiota changes were reverted after the 14-day washout*. Conclusion*. Some of the health-related benefits of omega-3 may be due, in part, to increases in butyrate-producing bacteria. These findings may shed light on the mechanisms explaining the effects of omega-3 in several chronic diseases and may also serve as an existing foundation for tailoring personalized medical treatments.

## 1. Introduction

Diets such as the Mediterranean, which is widely accepted as a healthy dietary pattern, have been promoted as a model of healthy eating based on its strong association with preserving a good health status and quality of life [1]. As a healthy dietary pattern the Mediterranean main food sources are characterized by high consumption of fruits, vegetables, grains, olive oil, and red wine (mainly served with food) as well as sea-fish on regular basis [1, 2]. Moreover, some of the marine sources of omega-3 polyunsaturated fatty acids (omega-3-PUFA) include eicosapentaenoic acid (EPA or 20 : 5), docosahexanoic acid (DHA, 22 : 6), and docosapentaenoic acid (DPA, 22 : 5), which are the longer chain n-3 forms [3]. Interestingly, high consumption of fish oils providing a major source of omega-3 (*ω*-3 or n-3) has been implicated as important contributors of the health-related benefits associated with the dietary pattern such as the Mediterranean.

Prior studies have documented the omega-3-PUFA-induced beneficial effects for a number of disorders including cardiovascular, neurodegenerative, neuropsychiatric, and inflammatory diseases, as well as for some cancer types (mainly colorectal, mammary, and prostatic cancer) [4]. For instance, a systematic review found that consumption of marine n-3 PUFA significantly lowered the risk of coronary heart disease death and sudden cardiac death [5]. Additionally, prospective cohort studies have examined the association between the consumption of either fish or fish oil supplements and breast cancer risk, showing a protective effect of omega-3 PUFA [6]. Although the underlying mechanisms accountable for the omega-3-PUFA-induced health effects are poorly understood, it appears that the impact of the omega-3 on the gut microbiota may play a pivotal role.

The relationship between the gut microbiota and its host plays a key role in immune system maturation, food digestion, drug metabolism, detoxification, vitamin production, and prevention of pathogenic bacteria adhesion. In fact, the composition of the microbiota is influenced by environmental factors such as diet, antibiotic therapy, and environmental exposure to microorganisms. Additionally, gut microbiota can vary according to sex, age, and geographical origin of the individual [7]. More importantly, an overgrowth of pathogenic microbial colonies may trigger an imbalance known as dysbiosis, a condition that has been implicated in the development of multiple diseases, such as cancer, metabolic diseases, and autoimmune conditions, and increased susceptibility to infection. The major taxa present in gut microbiota primarily consist of two major bacterial phyla,* Firmicutes* and* Bacteroidetes*, whose proportions appear to remain remarkably stable over time within individuals [8]. Some enterotypes are strongly associated with long-term diets, particularly protein and animal fat (*Bacteroides*) versus carbohydrates (*Prevotella*). However, the impact of omega-3 fatty acid rich diets on the gut microbiota and, more importantly, as a modulator of the bacterial populations associated with overall health has been poorly explored.

Since the gut microbiota represents a new target for therapeutic manipulation and prevention of multiple diseases while diet is considered to be a major environmental factor influencing gut microbiota diversity and functionality, we therefore present a case illustrating the effect of a diet rich in omega-3 fatty acids on the gut microbiota. Understanding the impact of dietary interventions on the gut microbiota may have potential clinical implications for the development of targeted treatments aimed at specific medical entities including cardiometabolic disease, inflammatory conditions, and cancers.

## 2. Case Presentation

A 45-year-old apparently healthy physically active male, whose typical diet included red meat and vegetables, is presented in this case. The patient demographics include being Caucasian, weight 89.8 kg, height 1.78 m, and BMI 28.3 kg/m^2^. Patient changed to a fish protein only diet with vegetables that included over 600 mg of Omega-3 fatty acids daily. For gut microbiota assessment, fecal samples were taken prior to the diet (Before), at the end of the 2 week diet omega-3 rich diet, and 2 weeks after (Washout) transitioning back to a diet that included red meat. Fecal samples were aseptically swabbed onto Whatman FTA cards (GE Healthcare Life Sciences) using sterile swabs and gloves to avoid environmental contamination. Collected samples placed on FTA cards were placed into sterile pouches and stored at room temperature prior to DNA amplification.

### 2.1. DNA Extraction and Amplification

From the FTA cards, 2 mm circular punches were taken and then washed with FTA reagent and TE (10 mM Tris-HCl, 1 mM EDTA, pH 8.0) according to the manufacture's protocol and air-dried at room temperature. Dried punches were used as template DNA for thermal cycling. For thermal cycling and DNA amplification the 16S universal Eubacterial primers 27f 5′-AGAGTTTGATCCTGGCTCAG-3′ and 1492r primer 5′-ACGGCTACCTTGTTACGACTT-3′ (Integrated DNA Technologies) were used. To amplify the DNA a single-step 30-cycle PCR using EconoTaq PLUS 2x Master Mix (Lucigen, Meddleton, WI) were used under the following conditions: 94°C for 2 minutes, followed by 30 cycles of 95°C for 120 seconds; 42°C for 30 seconds and 72°C for 4 minutes, after which a final elongation step at 72°C for 120 minutes was performed. Following PCR, the DNA products were resolved in a 1% agarose, 1x TAE gel stained with ethidium bromide. The 1.5 Kb DNA products were excised from the gel and purified using a cyclo-prep spin column (Amresco, Solon, OH). All DNA products were purified using Agencourt Ampure beads (Agencourt Bioscience Corporation, MA, USA).

### 2.2. Ion Torrent PGM Sequencing and Analysis

For ion torrent PGM sequencing, the 16S rRNA V4 variable region PCR primers 515/806 were used in a single-step 30 cycle PCR using the HotStarTaq Master Mix Kit (Qiagen, USA) with the following conditions: 94°C for 3 minutes, followed by 28 cycles of 94°C for 30 seconds, 53°C for 40 seconds, and 72°C for 1 minute, concluding with a final elongation step at 72°C for 5 minutes. The sequencing was performed at MR DNA (http://www.mrdnalab.com/, Shallowater, TX, USA) on an Ion Torrent PGM as previously described [9, 10]. The subsequent data were processed using a proprietary analysis pipeline (MR DNA, Shallowater, TX, USA). Sequences were depleted of barcodes and primers. Chimeric sequences, fragments shorter than 150 bp, sequences with ambiguous base call, and homopolymer runs exceeding 6 bp were removed. Operational taxonomic units (OTUs) were defined by clustering at 3% divergence (97% similarity). Conclusive OTUs were taxonomically classified using BLASTn against a curated GreenGenes database [11].

### 2.3. Impact of Omega-3 Rich Diet on the Gut Microbiota

**Figure 1:**
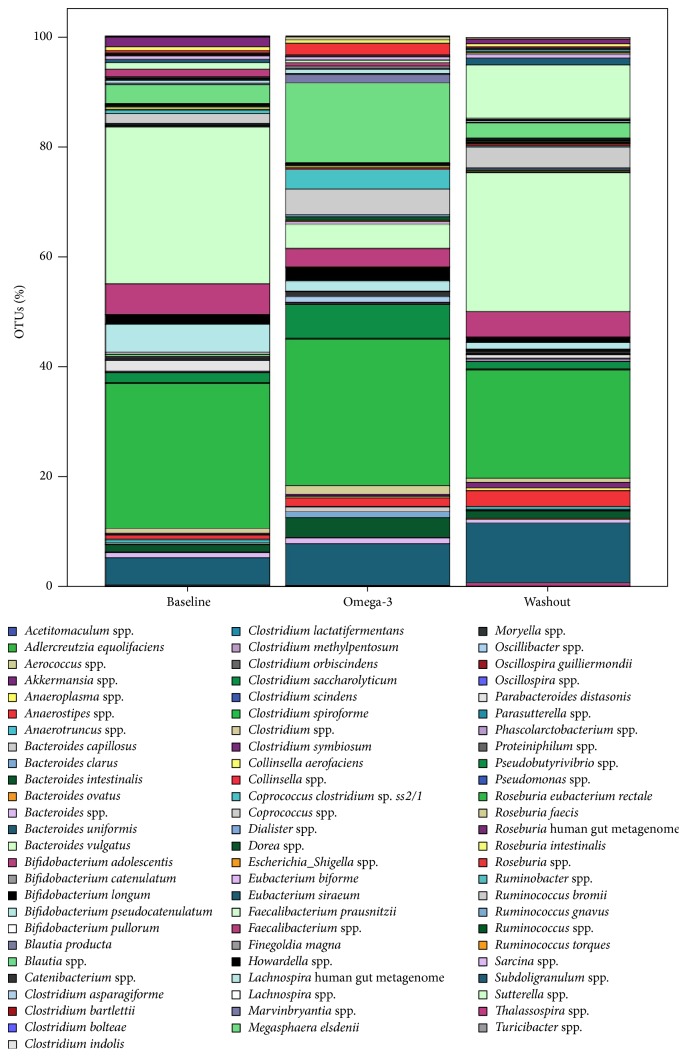
Variations of the bacterial species in the gut microbiota at baseline, after omega-3 rich diet, and at washout. OTUs: operational taxonomic units.

**Figure 2:**
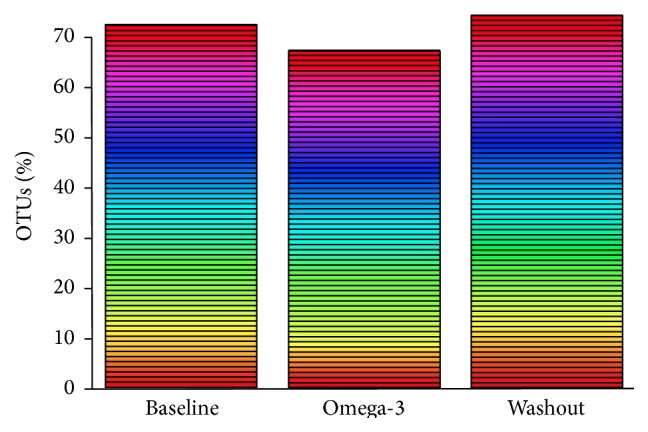
Rarefaction curve of the gut microbiota species at baseline, after omega-3 rich diet, and at washout. OTUs: operational taxonomic units.

Taxonomy-based analysis showed that the predominance of the major phyla did not change in response to the omega-3 rich diet (Figure 1) although the bacterial diversity was minimally reduced in response to the omega-3 rich diet (Figure 2). The main dominant phyla identified were Firmicutes, Bacteroidetes, and Actinobacteria. The phylum Firmicutes increased (Before versus omega-3: 89.52% versus 95.49%) with the omega-3 rich diet, but the phyla Bacteroidetes and Actinobacteria decreased (4.62% versus 1.23% and 3.15% versus 2.75%, resp.). After 2 weeks, however, of non-fish animal meat diet, there was a dramatic expansion in the phylum Bacteroidetes (1.23% versus 13.27%) and a concurrent reduction in the phylum Firmicutes (95.49% versus 83.23%) suggesting that the gut microbiota is a dynamic ecosystem susceptible to diet changes.

**Figure 3:**
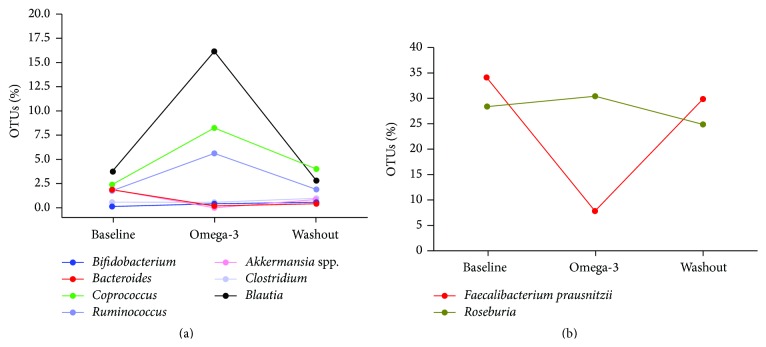
((a) and (b)) Variations of the major bacterial genera in the gut microbiota at baseline, after omega-3 rich diet, and at washout. OTUs: operational taxonomic units.

At the genus level, the 5 most abundant genera in the Before sample were* Faecalibacterium*,* Roseburia*,* Lachnospira*,* Subdoligranulum,* and* Blautia*.* Faecalibacterium prausnitzii* was the predominant species (Figure 3). At the end of the 2-week omega-3 rich diet, we identified a striking reduction in* Faecalibacterium*, and a remarkable increase in* Blautia* (3.75% versus 16.16%),* Coprococcus* (2.42% versus 8.25%),* Ruminococcus* (1.76% versus 5.60%), and* Subdoligranulum* (4.93% versus 7.57%).* Roseburia eubacterium rectale* became the predominant species. After 2 weeks of washout, the most remarkable changes in the microbiota were the expansion in the genera* Faecalibacterium* (7.80% versus 29.92%) and* Bacteroides* (1.11% versus 12.62%) and the reduction in* Blautia*,* Roseburia*,* Ruminococcus,* and* Coprococcus*.

## 3. Discussion

We sought to understand the changes of the gut microbiota in response to an omega-3 rich diet in a healthy male adult. The patient's predominance of the major phyla did not change with omega-3. At a genus level, however, there was a temporal shift in the composition of microbial communities, with a substantial increase in* Blautia* and a remarkable reduction in* Faecalibacterium* at the end of the 2-week omega-3 rich diet. Furthermore, by the end of the 2-week washout, the genera* Faecalibacterium* and* Bacteroides* expanded again. Taken together, these findings demonstrate that an omega-3 rich diet is capable of producing significant changes in the gut microbiota, which may explain its health benefits in several chronic diseases.

The adult colonic and fecal microbiota is dominated by obligate anaerobes with* Firmicutes* and* Bacteroidetes* together representing more than 80%, followed by* Actinobacteria*,* Proteobacteria*, and* Verrucomicrobia*, which are frequent, but generally minor constituents. Despite the consistency of these major components, their relative proportions and the species present vary dramatically between individuals [12, 13]. Accordingly,* Firmicutes* and* Bacteroidetes* together represented 95% of the gut microbiota in this patient and were kept as the major phyla despite diet modifications. On the other hand, we found important variations at a genus level, suggesting that omega-3 may impact gut microbiota at a genus/species level rather than a phylum level.

Of special interest is the degradation of otherwise nonfermentable dietary fiber such as resistant starch into short-chain fatty acids (SCFAs), mainly by bacteria from the Bacteroidetes and Firmicutes phyla. Acetate, propionate, and butyrate are the three major SCFAs in the colon. These SCFAs are an energy source for epithelial cells and have anti-inflammatory and immune-signaling properties [14]. Butyrate-producing bacteria (e.g.,* Faecalibacterium prausnitzii*,* Eubacterium rectale/Roseburia *spp.) represent a functional group, rather than a coherent phylogenetic group, within the microbial community of the human gut microbiota [15]. We found a remarkable increase in the genera* Eubacterium*,* Roseburia*,* Anaerostipes*,* Coprococcus*,* Subdoligranulum*, and* Pseudobutyrivibrio* after 2 weeks of the omega-3 rich diet, which are genera associated with butyrate production. Butyrate plays a key role in maintaining human gut health, as the major source of energy to the colonic mucosa and as an important regulator of gene expression, inflammation, differentiation, and apoptosis in host cells [15]. It appears that omega-3 can increase some butyrate-producing bacteria suggesting that omega-3 could benefit patients with noncommunicable chronic diseases.

Increasingly, the intestinal microbiota is recognized as an important player in human illness such as colorectal cancer (CRC). The composition of the gut bacteria community is different between healthy individuals and colon cancer patients. Studies have shown that several butyrate-producing bacterial genera were underrepresented in the stool of CRC patients compared to healthy individuals. The mechanisms by which bacteria contribute to CRC are complex and not fully understood, but increasing evidence suggests a link between the intestinal microbiota and CRC as well as diet and inflammation [16]. A study in animal models of colorectal cancer found a significant reduction of* Roseburia* and* Eubacterium* [17]. Additionally, reductions in* Blautia* were associated with increased incidence of colorectal cancer in both humans and mice [18]. In this patient, we found a remarkable increase in these genera after the omega-3 rich diet. These findings suggest that omega-3 might be useful in colorectal cancer treatment as it increases colon healthy bacterial populations.

Changes in the microbiota, and consequently in SCFAs composition, have also been hypothesized to be associated with the development of obesity, insulin resistance, and diabetes. Besides acting as a local nutrient source, SCFAs can also trigger cell-specific signaling cascades by receptor activation which may be involved in several positive effects. Moreover, omega-3 rich dietary seems to promote health by decreasing inflammatory state that reduces insulin resistance, increasing GLP-1 secretion that stimulates insulin release, and improving beta-cell function [19]. For instance, increased levels of* Roseburia* were associated with improved insulin sensitivity after gut microbiota transplantations from lean donors to recipients with metabolic syndrome while other butyrate-producing bacteria seem to play an important role in blood glucose regulation and lipid metabolism, as shown by fecal transplantation studies [19]. As we previously described, we found a remarkable increase in some butyrate-producing species after 2 weeks of the omega-3 rich diet. The fascinating role of gut microbiota in metabolic disease opens new directions in the treatment of obesity and insulin resistance, and omega-3 could play an important role as a gut microbiota modulator.

Although we found some positive changes in the gut microbiota after the omega-3 rich diet, we also observed an important reduction of the genus* Faecalibacterium*, specifically of* Faecalibacterium prausnitzii*. The reduction of* F. prausnitzii* in mucosal and fecal samples represents the most replicated species-specific finding so far in Crohn's disease (CD) [20]. Indeed, the risk of recurrence of CD following surgical resection was reported to be increased in patients whose mucosal* F. prausnitzii* populations were low [15]. This finding supports the idea that omega-3 is probably ineffective for maintenance of remission in CD, as it was noticed in a Cochrane systematic review [3].

Studies have reported conflicting conclusions about the benefits of omega-3 in diabetes. A recent meta-analysis found that marine omega-3 fatty acids increased risk of type 2 diabetes mellitus (T2DM) in Americans but reduced the same in Asians. Possible explanations for the difference in effect of omega-3-PUFA on different populations is the influence of genes and gene diet interaction and the differences in dietary patterns between Asian and Western populations [21]. Perhaps intestinal dysbiosis could also explain these contradictory findings. Recently, some studies reported that subjects with T2DM had a lower proportion of butyrate-producing Clostridiales (*Roseburia* and* Faecalibacterium prausnitzii*) and greater proportions of Clostridiales that do not produce butyrate [22]. Another study found that subjects with higher gene richness and* Akkermansia muciniphila* abundance displayed greater improvement in insulin sensitivity markers. Abundance of* Akkermansia muciniphila*, a mucin-degrading bacterium, has been inversely associated with body fat mass and glucose intolerance in mice [23]. Additionally, in an experimental mice study, it was demonstrated that metformin is able to affect the mouse microbiota and increase the abundance of* Akkermansia muciniphila* [22]. In our patient, we observed an important reduction in* Akkermansia *spp. after 2 weeks of the omega-3 rich diet.

The main limitation of the present case is that our findings were obtained from a single patient and therefore may be considered preliminary. Another potential imitation is that the patient was not tracked with a dietary journal. However, dietary journals have been consistently recognized as unreliable especially in the absence of suitable biomarkers. This case argues in favor of using of the gut microbiota as a reliable biomarker for monitoring diet adherence during therapeutic interventions [24]. There is, however, the need for a large number of patients and future replication of these results.

In sum, consumption of omega-3 rich diets has been postulated beneficial for health, but the gut microbiota changes associated with omega-3 fatty acids are poorly understood. Here we report significant changes in the gut microbiota after an omega-3 rich diet in bacterial populations associated with health. In addition, the apparent lack of benefit in response to omega-3 associated with certain disorders such as CD was explored suggesting that this dietary pattern is not suited for all clinical populations. The findings presented in this case may shed light on the underlying mechanisms explaining beneficial effects of omega-3 in several chronic diseases and may also serve as an existing foundation for tailoring personalized medical treatments.
